# Sarcopenic obesity and reduced BMD in young men living with HIV: body composition and sex steroids interplay

**DOI:** 10.1007/s40618-024-02375-6

**Published:** 2024-04-20

**Authors:** S. De Vincentis, C. Greco, F. Fanelli, M. C. Decaroli, C. Diazzi, M. Mezzullo, J. Milic, M. C. De Santis, L. Roli, U. Pagotto, G. Guaraldi, V. Rochira

**Affiliations:** 1 Unit of Endocrinology, Department of Biomedical, Metabolic and Neural Sciences, University of Modena and Reggio Emilia, Via Giardini, 1355, 41126 Modena, Italy; 2 Unit of Endocrinology, Department of Medical Specialties, Azienda Ospedaliero-Universitaria of Modena, Modena, Italy; 3 Endocrinology Research Group, Department of Medical and Surgical Sciences, Center for Applied Biomedical Research, Alma Mater Studiorum University of Bologna, Bologna, Italy; 4 Multidisciplinary Metabolic Clinic, Unit of Infectious Diseases, University of Modena and Reggio Emilia, Modena, Italy; 5 Department of Laboratory Medicine and Pathology, Azienda USL of Modena, Modena, Italy; 6 Division of Endocrinology and Diabetes Prevention and Care, IRCCS Azienda Ospedaliero-Universitaria Di Bologna, Bologna, Italy

**Keywords:** Sarcopenia, Visceral fat, Estrogen, Androgen, Estradiol/testosterone ratio, Comorbidity, Bone mass

## Abstract

**Purpose:**

Sex steroids play a key role on male bone homeostasis and body composition (BC), their role in men living with HIV (MLWH) is less recognized. This study aimed at investigating the prevalence of low BMD, sarcopenia, and sarcopenic obesity (SO) and their relationship with sex steroids in MLWH aged < 50.

**Methods:**

Prospective, cross-sectional, observational study on MLWH younger than 50 (median age 47.0 years). BC and BMD were evaluated with DXA. Two different definitions of sarcopenia were applied: appendicular lean mass/height^2^ (ALMI) < 7.26 kg/m^2^ or appendicular lean mass/body weight (ALM/W) < 28.27%. Low BMD was defined for *Z*-score < −2.0. Sarcopenia coupled with obesity identified SO. Serum total testosterone (T) and estradiol (E2) were measured by LC–MS/MS; free testosterone (cFT) was calculated by Vermeulen equation.

**Results:**

Sarcopenia was detected in 107 (34.9%) and 44 (14.3%) out of 307 MLWH according to ALMI and ALM/W, respectively. The prevalence of SO was similar by using both ALMI (11.4%) and ALM/W (12.4%). Sarcopenic and SO MLWH had lower total T and cFT in both the definition for sarcopenia. BMD was reduced in 43/307 (14.0%). Serum E2 < 18 pg/mL was an independent contributing factor for sarcopenia, SO, and low BMD.

**Conclusions:**

T and E2 are important determinants of BC even in MLWH. This is among the first studies investigating the distribution of obesity phenotypes and the prevalence of SO among MLWH showing that SO is present in 11–12% of enrolled MLWH regardless of the definition used. However, deep differences emerged using two different diagnostic definitions.

## Introduction

The clinical picture of men living with HIV (MLWH) has notably evolved thanks to the improvement in antiretroviral therapies (ART) [1, 2]. As life expectancy in MLWH has increased [3], the clinical management of HIV has moved from the sole control of the disease also to the prevention and treatment of non-infectious comorbidities related to ART and aging [4], and sustain of health related quality of life. Many of these chronic comorbidities are frequent even in the general population but they prematurely occur in MLWH [5, 6].

Hypogonadism, osteoporosis, high risk of fractures, and changes in body composition are common HIV-related comorbidities [7–12]. Otherwise, sex steroids, particularly testosterone (T) and estradiol (E2), are factors able to strongly influence bone mass and body composition [13–17]. Beyond low serum T and low BMD, changes in body composition have been linked to HIV infection and treatment since the ‘80s. Even though new ARTs have fewer adverse effects and the magnitude of lipodystrophy appears to have decreased [18], sarcopenia, defined as reduced muscle mass, continues to be reported in MLWH [19, 20]. Additionally, sarcopenic obesity is a new construct of obesity in older adults who have high adiposity coupled with low muscle mass. Recent reviews have highlighted the impact of sarcopenic obesity on general health outcomes, cardiometabolic impairment, and mortality in the general population [21], but data in MLWH are scanty [22]. Furthermore, diagnostic criteria for sarcopenia changed throughout the years and different operational definitions are currently available; to date, there is no global consensus on which criteria to use for both sarcopenia and sarcopenic obesity even though consensus on this issue are becoming available [23, 24].

Although the etiology of such gonadal and musculoskeletal changes remains partly understood, either hypogonadism, osteoporosis, or sarcopenia maybe strictly interrelated in MLWH [8, 9]. As a matter of fact, androgen exposure (of which serum T levels might reflective) be directly associated with bone tissue, fat and lean mass in MLWH [25] and low BMD and sarcopenia are correlated in both men and women [26]. In line with this findings, emerging evidence confirms the mutual relationship among comorbidities, health status, and sex steroids in MLWH [25, 27, 28] as well as in male general population [29–32]. New insights have been recently added in this topic proposing HIV-related hypogonadism as a form of functional hypogonadism secondary to frailty and chronic comorbidities [25, 28]. However, the vicious circle between gonadal function, body composition and bone health remains to be fully elucidated in MLWH, especially in the young-to-middle age. With this in view, the aim of this study was to prospectively explore the relationship between serum sex steroids by the gold standard technology, body composition, and bone health status in a cohort of MLWH younger than 50 years. Secondary aims were to report the prevalence of sarcopenia, sarcopenic obesity and low BMD and to detect possible factors associated with these conditions.

## Methods

### Study design and participants

This was a cross-sectional observational study of prospectively collected data from May 2013 to December 2017, enrolling MLWH attending the Modena HIV Metabolic Clinic (MHMC) as previously described [25].

Inclusion criteria were a documented HIV-infection on ART treatment, age 18–50 years and written informed consent. In order to avoid the overlap with hypogonadism due to aging, the limit of age for enrolment was per protocol fixed below or equal to 50 years. Exclusion criteria were: severe liver or renal insufficiency, active malignancy, acquired immunodeficiency syndrome (AIDS), endocrine disease (pituitary, thyroid, testicular diseases), any medication that could interfere with gonadal function as well as any previous treatment involving pituitary region (surgery, radiotherapy).

A total of 319 MLWH were assessed for eligibility, 3 were excluded from the study (1 patient with severe liver insufficiency; 1 patient with sever chronic kidney disease; 1 patient with prior androgen treatment). Finally, 316 patients were enrolled.

### Main outcome measures

#### Anthropometric evaluation and body composition assessment

Anthropometric measurements (weight, height, body mass index [BMI], waist, hip, and waist/hip ratio) were recorded at the time of the examination. Weight was measured after at least a 5-h fast by analogical balance and height by stadiometer. Waist and hip circumferences were measured as the average of 3 measurements. BMI was calculated as body weight (kilograms) divided by the square of height (square meters). A whole body composition assessment and measurement of BMD were both obtained by Dual-Energy X-ray Absorptiometry (DXA) (DXA, Hologic-QDR-2000 densitometer, Inc., Waltham, MA) according to standardized procedures [33, 34]. Precision error rates for the QDR 2000 were of 3% for the measurement of fat mass, and 1.5% for fat-free mass. DEXA underwent calibration daily to ensure accurate BMD measurements using an anthropometric phantom.

As a consensus for defining sarcopenia is lacking, we adopted two different operational definitions to diagnose sarcopenia: i) ALMI: appendicular lean mass index (appendicular lean mass/height^2^) with a cut off for sarcopenia of < 7.26 kg/m^2^ according to Baumgartner’s criteria [35]; ii) ALM/W: appendicular lean mass/body weight with a cut off for sarcopenia of < 28.27% as suggested by the recent European consensus statement [23, 36]. While ALMI is currently the most used index in the setting of MLWH [37], the application of ALM/W is catching on for the definition of sarcopenia in the general population [23]. DXA parameters were missing in 9 patients that were excluded from the analysis.

Obesity was defined as BMI ≥ 30 kg/m^2^ [38] and/or fat mass > 26% in men aged 20–39 and > 28% in men aged 40–50 [39]. According to BMI, men were further classified as underweight (< 18.5 kg/m^2^) and normal (18.5–25 kg/m^2^). Based on the combination of BMI and body fat mass, patients were classified into the following obesity phenotypes: i) normal: BMI 18.5–25 kg/m^2^ and body fat percentage < 26% or < 28% depending on patients’ age; ii) overweight: BMI 25–30 kg/m^2^ and body fat percentage < 26% or < 28% depending on patients’ age; iii) hidden obese: BMI 18.5–30 kg/m^2^ and body fat percentage > 26% or > 28% depending on patients’ age; iv) overt obese: BMI ≥ 30 kg/m^2^ and body fat mass > 26% or > 28% depending on patients’ age; v) underweight: BMI < 18.5 kg/m^2^.

Finally, sarcopenic obesity was defined by the coupled presence of sarcopenia and obesity, each defined as described above [23, 36, 39].

#### BMD assessment

BMD was assessed by the same DXA methodology described above. BMD was measured at lumbar spine (L1 to L4) and hip (total and neck). Precision error rates for the QDR 2000 were 1% or less for BMD. DEXA underwent calibration daily to ensure accurate BMD measurements using an anthropometric phantom. BMD measured by DXA is expressed as an areal density in g/cm^2^. For age < 50 years a condition of low BMD was defined for *Z*-score < 2.0 at lumbar or femoral site.

#### Laboratory analyses

Blood samples were collected at 8:00 am after an overnight fasting by an intravenous cannula inserted into an antecubital vein. The blood samples were centrifuged, and the serum was stored at −20 °C until assayed.

All biochemical and hormonal measurements have been performed as previously described [25]. In detail, sex steroids (total T, E2, estrone [E1], and dihydrotestosterone [DHT]) were assayed by validated liquid chromatography-tandem mass spectrometry technique (LC–MS/MS) methods [40–42]. Of the 316 enrolled patients, 71 participants were missing E2 data. Free T was calculated (cFT) by using the validated Vermeulen equation [43, 44].

Sex Hormone-binding globulin (SHBG), serum luteinizing hormone (LH), follicle stimulating hormone (FSH), and prolactin (PRL) were assessed by chemiluminescent immunoassay, as previously described [25].

#### Demographic and clinical parameters

At the moment of the visit, all participants answered a structured questionnaire. The questionnaire contained demographic data (age) and clinical data (date of HIV diagnosis, history of fractures, history of comorbidities, medications in use, physical activity, smoking, and alcohol intake). Data related to HIV infection were obtained from the medical records of the participants.

For the definition of frailty index (FI), please refer to previous publications [25, 45].

### Statistical analysis

The non-parametric Mann–Whitney *U* test was used for comparisons of continuous variables since they resulted not normally distributed at the Kolmogorov–Smirnov test. Categorical variables were compared by Pearson’s Chi-squared test.

Linear regression was used to examine the association between continuous variables; significant results were expressed through the *R*^*2*^ coefficient.

Ordinal logistic regression was used to determine contributing factors to sarcopenia, low BMD, and sarcopenic obesity. Proportional odds models were used to estimate the odds ratio (OR) for the risk/protective factors.

Statistical analyses were performed using the Statistical Package for the Social Sciences’ (SPSS) software for Windows (version 28.0; SPSS Inc, Chicago, IL). For all comparisons, *p* < 0.05 was considered statistically significant.

## Results

A total of 307 consecutive MLWH were enrolled, with a median age of 47.0 years (range 25.2–50.5 years) and a median duration of HIV-infection of 16.2 years (range 1.1–35.4 years). For comprehensive characteristics of the 316 patients, see our previous publication [25].

### Prevalence of sarcopenia and associated factors

Sarcopenia was detected in 107 (34.9%) and 44 (14.3%) MLWH according to ALMI and ALM/W, respectively (Table 1).

**Table 1:** Differences in clinical, biochemical and body composition parameters comparing MLWH with sarcopenia to those non-sarcopenic according to two different diagnostic criteria for defining sarcopenia: ALMI ≤ 7.26 kg/m^2^ and ALM/W < 28.27%

	n.v	MLWH with sarcopenia as ALMI < 7.26 kg/m^2^	MLWH without sarcopenia as ALMI ≥ 7.26 kg/m^2^	*p*-value	MLWH with sarcopenia as ALM/W < 28.27%	MLWH without sarcopenia as ALM/W ≥ 28.27%	*p*-value
*n 307*		107 (34.9%)	200 (65.1%)		44 (14.3%)	263 (85.7%)	
Age (years)	–	46.7 (41.0–49.6)	47.2 (43.0–49.8)	0.368	47.4 (43.2–49.9)	46.8 (42.4–49.7)	0.582
Anthropometric variables							
BMI (kg/m^2^)	18.5–25	22.1 (20.5–23.5)	24.5 (22.8–26.3)	** < 0.001**	26.2 (23.5–29.3)	23.5 (21.6–29.1)	** < 0.001**
W/H circumference ratio	< 0.95	0.95 (0.88–0.99)	0.95 (0.90–0.98)	0.354	0.99 (0.96–1.04)	0.94 (0.89–0.98)	** < 0.001**
HIV parameters							
HIV duration (years)	–	15.9 (8.0–25.4)	16.2 (9.2–23.3)	0.753	13.9 (8.8–25.3)	16.3 (8.4–23.5)	0.995
ART exposure (years)	–	14.2 (5.5–21.0)	14.3 (7.0–18.9)	0.957	12.9 (7.7–17.9)	14.5 (6.3–19.9)	0.824
Nadir CD4 (cells/μL)	–	285 (218–418)	224 (87–331)	0.056	267 (125–412)	250 (99–345)	0.643
Current CD4 (cells/μL)	> 400	621 (525–863)	607 (505–772)	0.448	629 (518–720)	619 (510–829)	0.663
Hormonal measurements							
LH (mIU/mL)	1.4–8.9	4.9 (3.8–7.4)	4.9 (3.5–6.6)	0.257	4.3 (3.3–6.7)	4.9 (3.6–6.8)	0.540
FSH (mIU/mL)	1.7–6.9	6.4 (4.0–8.7)	5.4 (3.8–8.1)	0.165	6.1 (4.1–8.7)	5.6 (3.8–8.2)	0.340
PRL (ng/mL)	2.1–17.7	7.8 (5.8–11.1)	7.2 (5.1–9.2)	0.076	7.6 (5.1–9.8)	7.4 (5.3–9.8)	0.717
Serum E2 (pg/mL)	< 50	20.5 (17.5–29.0)	26.2 (20.9–31.8)	0.145	19.3 (15.3–34.1)	24.8 (20.3–31.1)	0.205
Serum E1 (pg/mL)		25.0 (16.5–34.4)	29.0 (20.3–37.5)	0.063	29.1 (15.6–46.1)	27.6 (19.0–37.1)	0.967
Serum TT (ng/dL)	> 320	624 (498–790)	644 (514–798)	**0.002**	545 (415–690)	657 (524–807)	**0.001**
E2/TT	–	0.035 (0.028–0.042)	0.039 (0.031–0.048)	**0.017**	0.038 (0.029–0.053)	0.038 (0.030–0.047)	0.709
SHBG (nmol/L)	13.5–71.4	58.7 (30.9–70.6)	45.6 (33.9–65.4)	**0.006**	52.5 (35.5–70.6)	47.8 (35.2–65.0)	0.363
cFT (pg/mL)	> 64	98 (76–121)	110 (90–135)	**0.002**	87 (67–107)	110 (89–135)	** < 0.001**
DHT (pg/mL)	165–679	431 (276–560)	372 (261–527)	0.188	353 (231–479)	382 (264–547)	0.361
Biochemical measurements							
Fasting glucose (mg/dL)	70–100	92 (86–96)	93 (87–100)	0.210	93 (87–100)	92 (86–98)	0.466
Insulin (mIU/mL)	2–23	6.6 (4.7–11.3)	8.3 (5.1–13.4)	**0.022**	10.0 (6.2–13.5)	7.2 (4.8–12.3)	**0.015**
HOMA index	< 2.5	1.48 (1.02–2.79)	1.87 (1.17–3.12)	**0.017**	2.32 (1.33–3.18)	1.67 (1.08–2.98)	**0.017**
HbA1c (mmol/mol)	20–38	33.0 (31.1–36.0)	34.0 (31.1–36.6)	0.163	34.0 (30.3–36.8)	34.0 (31.0–36.6)	0.824
Total cholesterol (mg/dL)	< 200	181 (159–215)	190 (165–218)	0.087	189 (158–218)	188 (165–218)	0.868
LDL cholesterol (mg/dL)	< 100	112 (87–135)	120 (100–144)	**0.028**	118 (93–141)	118 (97–141)	0.937
HDL cholesterol (mg/dL)	> 45	47 (38–52)	45 (37–55)	0.521	44 (35–51)	47 (37–55)	0.280
Triglycerides (mg/dL)	< 180	126 (83–184)	131 (91–198)	0.272	147 (92–188)	128 (86–191)	0.368
Lifestyle and drug use							
Smoking	–	60 (56.6%)	62 (31.5%)	** < 0.001**	23 (53.5%)	99 (38.9%)	0.059
Alcohol use (moderate/intense)	–	61 (57.5%)	103 (52.0%)	0.357	26 (60.5%)	138 (56.1%)	0.368
Opioids use	–	22 (20.8%)	42 (21.2%)	0.926	8 (18.6%)	55 (21.2%)	0.703
Comorbidities and frailty							
Diabetes	–	2 (1.9%)	5 (2.5%)	0.724	1 (2.3%)	6 (2.3%)	0.994
Hypertension	–	17 (16.0%)	36 (18.2%)	0.639	10 (23.3%)	43 (16.5%)	0.283
Cardiovascular disease	–	7 (6.6%)	12 (6.1%)	0.852	3 (7.0%)	16 (6.2%)	0.837
Dyslipidemia	–	49 (46.7%)	99 (50.0%)	0.581	19 (45.2%)	128 (49.2%)	0.631
HCV/HBV co-infection	–	20 (18.9%)	37 (18.6%)	0.670	7 (16.3%)	50 (19.0%)	0.602
Previous atraumatic fractures	–	1 (0.9%)	4 (2.0%)	0.482	1 (2.3%)	4 (2.5%)	0.707
Frailty index	< 0.21	0.24 (0.18–0.31)	0.26 (0.19–0.33)	0.241	0.31 (0.25–0.37)	0.24 (0.18–0.32)	** < 0.001**
Bone and body composition parameters at DXA							
Lumbar BMD (g/cm^2^)	–	0.94 (0.88–1.01)	0.97 (0.90–1.05)	**0.018**	0.96 (0.89–1.03)	0.99 (0.89–1.06)	0.370
Total hip BMD (g/cm^2^)	–	0.81 (0.74–0.88)	0.87 (0.79–0.97)	** < 0.001**	0.85 (0.79–0.98)	0.84 (0.77–0.95)	0.573
Neck hip BMD (g/cm^2^)	–	0.74 (0.68–0.82)	0.80 (0.74–0.88)	** < 0.001**	0.76 (0.68–0.87)	0.78 (0.72–0.86)	0.374
Low BMD, *n* (%)	–	16 (15.1%)	27 (13.5%)	0.703	6 (14.0%)	35 (13.4%)	0.916
Lean mass—arms (kg)	–	5.78 (5.01–6.36)	7.29 (6.64–8.07)	** < 0.001**	5.56 (4.80–6.25)	6.93 (6.14–7.77)	** < 0.001**
Lean mass—trunk (kg)	–	24.0 (21.9–25.9)	25.9 (24.3–27.8)	** < 0.001**	26.4 (22.0–29.6)	25.3 (23.5–29.7)	0.207
Fat mass—trunk (kg)	–	8.4 (6.4–11.8)	8.5 (6.6–11.5)	0.477	14.4 (12.1–17.2)	7.9 (6.3–10.4)	** < 0.001**
Lean mass—legs (kg)	–	15.0 (13.9–16.2)	17.7 (16.3–18.8)	** < 0.001**	15.8 (14.0–18.6)	16.9 (15.4–18.2)	0.135
Lean mass—total body (kg)	–	49.3 (44.8–52.1)	55.0 (51.9–58.8)	** < 0.001**	51.4 (44.9–58.7)	53.1 (49.9–56.7)	0.261
Lean mass—total body (%)	–	72.9 (68.5–77.8)	73.6 (70.8–76.4)	0.492	64.4 (60.5–67.2)	73.7 (71.0–77.0)	** < 0.001**
Fat mass—total body (kg)	–	17.4 (13.3–20.9)	17.2 (13.8–21.6)	0.483	26.9 (21.3–32.1)	15.9 (13.2–19.7)	** < 0.001**
Fat mass—total body (%)	< 26% or < 28%	25.5 (21.1–29.6)	23.4 (20.0–27.5)	**0.038**	33.2 (30.1–35.8)	23.0 (19.9–26.4)	** < 0.001**
Fat mass—total body > 26% in aged 20–39 and > 28% in aged 40–50, *n* (%)	–	35 (32.7%)	45 (22.5%)	0.052	38 (86.4%)	(42 (16.0%)	** < 0.001**
ALMI (kg/m^2^)	> 7.26	6.68 (6.34–7.07)	8.15 (7.68–8.52)	** < 0.001**	6.99 (6.20–7.71)	7.76 (7.11–8.33)	** < 0.001**
ALM/W (%)	> 28.27	30.6 (28.2–33.3)	33.5 (31.4–33.2)	** < 0.001**	26.8 (25.3–27.5)	33.2 (31.3–35.0)	** < 0.001**

Only significant comparisons are in bold. Continuous data are reported as median (IQR)

*MLWH* men living with HIV, *ALMI* appendicular lean mass index, *ALM/W* appendicular lean mass to body weight ratio, *IQR* interquartile range, *BMD* bone mineral density, *BMI* body mass index, *ART* antiretroviral therapy, *LH* luteinizing hormone, *FSH* follicle-stimulating hormone, *PRL* prolactin, *TT* total testosterone, *E1* estrone, *E2* estradiol, *SHBG* sex hormone-binding globulin, *cFT* calculated free testosterone, *E2/T* estradiol to testosterone ratio, *DXA* dual-energy X-ray absorptiometry

#### MLWH with sarcopenia by ALMI

MLWH with ALMI-sarcopenia presented with significantly lower BMI compared to those without sarcopenia (*p* < 0.001) (Table 1). As expected, patients with sarcopenia had lower lean mass assessed at arms, trunk, legs, and total body; notably, the amount of total body fat mass was higher in sarcopenia subgroup (*p* = 0.038) (Table 1).

Bone parameters significantly differed in relation to the presence of ALMI-based sarcopenia. First, BMD values were lower at any site (lumbar, total and neck hip, *p* range: < 0.001–0.018) in sarcopenic MLWH, although the prevalence of low BMD for age was similar comparing the 2 subgroups (Table 1). Hip BMD (total hip: *p* < 0.001, β = 0.222, *R*^2^ = 0.050; neck hip: *p* = 0.008, β = 0.152, *R*^2^ = 0.023) was directly associated with ALMI; no significant association was confirmed for lumbar BMD at the bivariate regression analysis.

Interesting differences were found comparing sex steroids between sarcopenic and non-sarcopenic subgroups. First, total T (*p* = 0.002), E2/T ratio (*p* = 0.017), and cFT (*p* = 0.002) were significantly lower in MLWH with sarcopenia than those without; conversely, SHBG was higher in sarcopenic MLWH (*p* = 0.006) (Table 1). No difference was found for levels of estrogens, gonadotropins, and DHT (Table 1). At the bivariate linear regression analysis, only E2/T (*p* = 0.001, *β* = 0.235, *R*^2^ = 0.055) and SHBG (*p* = 0.017, *β* =−0.137, *R*^2^ = 0.019) were confirmed to be significantly associated with ALMI in opposite direction (directly for E2/T and negatively for SHBG); no significant regression was found for total T, cFT and E2 levels.

As to other biochemical data, HOMA index (*p* = 0.017) were slightly lower in MLWH with sarcopenia, whereas no difference was found for either fasting glucose or HbA1c levels (Table 1).

With regard to HIV-related data, no significant difference was found for HIV duration, ART exposure, CD4 nadir and current CD4 (Table 1). Finally, smoking was more frequent in sarcopenic than non-sarcopenic MLWH (*p* < 0.001) (Table 1).

A multivariate logistic regression analysis was performed to identify risk factors for sarcopenia defined by ALMI. Co-variates were chosen including variables that at univariate analysis displayed *p*-value < 0.10. The multivariate analysis showed that higher BMI values were protective for sarcopenia (OR = 0.64; 95% CI 0.56–0.72; *p* < 0.001), whereas E2 below 18 pg/mL was an independent risk factor for sarcopenia (OR = 3.50; 95% CI 1.20–10.20; *p* = 0.022). On the contrary, SHBG, total T and E2/T ratio, years of HIV, BMD values, and smoking lost the statistical significance.

#### MLWH with sarcopenia by ALM/W

Patients with ALM/W-sarcopenia presented with significantly higher BMI and W/H ratio compared to those without sarcopenia (*p* < 0.001) (Table 1). As expected, patients with sarcopenia had lower lean mass at arms and total lean body percentage (*p* < 0.001); notably, the total amount and the percentage of total body fat mass together with the trunk fat mass were all higher in sarcopenia subgroup (*p* < 0.001) (Table 1).

Bone parameters did not differ in relation to the presence of sarcopenia at ALM/W. Accordingly, no significant association was found between ALM/W and either lumbar or hip BMD at the bivariate regression analysis.

The following changes in circulating sex steroids between MLWH with and without ALM/W-sarcopenia were found: serum total T (*p* = 0.001) and cFT (*p* < 0.001) were significantly lower in sarcopenic than non-sarcopenic MLWH while other sex hormones and SHBG did not differ between the two groups.

Among other biochemical parameters, insulin (*p* = 0.015) together with HOMA index (*p* = 0.017) were slightly lower in non-sarcopenic than sarcopenic MLWH, whereas no difference was found for neither fasting glucose nor HbA1c levels (Table 1).

With regard to HIV-related parameters, no significant difference was found for HIV duration, duration of ART exposure, CD4 nadir and current CD4 (Table 1).

No differences in terms of lifestyle factors, and comorbidities were found, whereas MLWH with sarcopenia reported a frailty index significantly worse than MLWH without sarcopenia (*p* < 0.001) (Table 1).

A multivariate logistic regression analysis was repeated to identify risk factors for ALM/W-based sarcopenia, higher BMI values were protective for sarcopenia (OR = 0.65; 95% CI 0.48–0.88; *p* = 0.006), whereas E2 below 18 pg/mL was an independent risk factor for (OR = 18.80; 95% CI 3.10–114.05; *p* = 0.002). On the contrary, total T, cFT, W/H, and frailty score did not enter in the model.

## Prevalence of sarcopenic obesity and distribution of obesity phenotypes

Based on the two different operational definitions of sarcopenia, the prevalence of sarcopenic obesity was almost the same: in detail 11.4% (35 out of 307 MLWH) according to ALMI and 12.4% (38 out of 307 MLWH) according to ALM/W (Table 2).

**Table 2:** Differences in sex steroids comparing MLWH with sarcopenic obesity to those without sarcopenic obesity according to two different diagnostic criteria for defining sarcopenia: ALMI ≤ 7.26 kg/m^2^ and ALM/W < 28.27%

	n.v	MLWH with sarcopenic obesity according to ALMI	MLWH without sarcopenic obesity according to ALMI	*p*-value	MLWH with sarcopenic obesity according to ALM/W	MLWH without sarcopenic obesity according to ALM/W	*p*-value
*n 307*		36 (11.7%)	271 (88.3%)		38 (12.4%)	269 (87.6%)	
Age (years)	–	43.7 (39.5–48.5)	47.3 (43.0–49.8)	**0.025**	47.4 (43.0–49.8)	46.9 (42.5–49.8)	0.777
Anthropometric variables							
BMI (kg/m^2^)	18.5–25	23.7 (22.2–25.2)	23.6 (21–8-25.5)	0.684	26.9 (23.8–30.1)	23.5 (21.7–25.0)	** < 0.001**
W/H circumference ratio	< 0.95	0.97 (0.90–1.01)	0.95 (0.90–0.98)	0.126	1.00 (0.96–1.05)	0.94 (0.89–0.98)	** < 0.001**
HIV parameters							
HIV duration (years)	–	11.4 (6.4–20.9)	16.7 (9.2–24.3)	**0.045**	13.4 (8.7–24.2)	16.4 (8.4–23.7)	0.640
ART exposure (years)	–	11.4 (5.2–16.7)	14.9 (6.8–20.2)	0.132	12.9 (7.5–17.9)	14.6 (6.4–19.9)	0.535
Hormonal measurements							
LH (mIU/mL)	1.4–8.9	4.1 (3.4–6.8)	5.0 (3.5–6.8)	0.393	4.3 (3.3–6.0)	5.0 (3.6–6.9)	0.220
FSH (mIU/mL)	1.7–6.9	5.5 (3.0–7.9)	5.6 (3.9–8.4)	0.302	5.7 (4.1–8.6)	5.6 (3.8–8.3)	0.861
PRL (ng/mL)	2.1–17.7	7.2 (5.2–11.1)	7.4 (5.3–9.7)	0.819	8.0 (6.1–10.7)	7.4 (5.3–9.7)	0.271
Serum E2 (pg/mL)	< 50	18.8 (13.5–21.8)	25.3 (20.4–31.6)	**0.001**	24.1–16.5–35.6)	24.8 (20.2–31.0)	0.550
Serum E1 (pg/mL)		18.1 (13.1–26.8)	18.9 (20.0–37.6)	**0.005**	33.0 (16.3–19.0)	27.5 (18.8–36.9)	0.429
Serum TT (ng/dL)	> 320	538 (437–760)	655 (523–798)	**0.017**	538 (406–656)	656 (524–805)	**0.001**
E2/TT	–	0.037 (0.027–0.045)	0.038 (0.030–0.047)	0.443	0.040 (0.030–0.053)	0.038 (0.030–0.047)	0.345
SHBG (nmol/L)	13.5–71.4	54.5 (35.3–64.1)	47.8 (35.5–66.0)	0.939	52.5 (34.2–70.5)	47.8 (35.6–65.1)	0.647
cFT (pg/mL)	> 64	88 (74–111)	108 (88–133)	**0.004**	88 (64–109)	110 (88–135)	** < 0.001**
DHT (pg/mL)	165–679	384 (244–499)	381 (265–599)	0.428	378 (217–460)	382 (267–550)	0.309
Biochemical measurements							
Fasting glucose (mg/dL)	70–100	92 (87–98)	92 (86–98)	0.852	92 (87–101)	92 (87–98)	0.487
Insulin (mIU/mL)	2–23	9.2 (5.7–12.9)	7.4 (5.0–12.6)	0.512	10.2 (6.1–12.5)	7.3 (4.9–12.1)	**0.014**
HOMA index	< 2.5	2.17 (1.24–3.06)	1.69 (1.11–2.98)	0.541	2.35 (1.31–4.73)	1.68 (1.08–2.95)	**0.016**
HbA1c (mmol/mol)	20–38	33.0 (31.1–36.0)	34.0 (31.1–36.6)	0.362	34.0 (31.0–37.0)	34.0 (31.0–36.6)	0.448
Total Cholesterol (mg/dL)	< 200	211 (161–230)	186 (164–213)	0.156	190 (166–223)	187 (164–216)	0.512
LDL Cholesterol (mg/dL)	< 100	120 (94–160)	118 (97–140)	0.541	123 (99–145)	118 (97–141)	0.556
HDL Cholesterol (mg/dL)	> 45	47 (38–51)	45 (37–55)	0.791	44 (35–51)	46 (37–55)	0.256
Triglycerides (mg/dL)	< 180	141 (79–189)	129 (89–191)	0.916	156 (105–193)	126 (85–190)	0.102
Comorbidities and frailty							
Diabetes	–	0 (0%)	7 (2.6%)	0.334	1 (2.7%)	6 (2.2%)	0.863
Hypertension	–	5 (17.1%)	47 (17.5%)	0.961	9 (24.3%)	44 (16.5%)	0.239
Cardiovascular disease	–	3 (8.6%)	16 (5.9%)	0.546	3 (8.1%)	16 (6.0%)	0.618
Dyslipidemia	–	16 (47.1%)	132 (49.1%)	0.825	18 (50.0%)	130 (48.7%)	0.883
HCV/HBV co-infection	–	4 (11.4%)	53 (19.6%)	0.242	5 (13.2%)	52 (19.5%)	0.350
Previous atraumatic fractures	–	1 (2.9%)	4 (1.5%)	0.549	1 (2.73%)	4 (1.5%)	0.589
Frailty index	< 0.21	0.25 (0.16–0.31)	0.25 (0.19–0.32)	0.510	0.32 (0.25–0.38)	0.24 (0.18–0.32)	** < 0.001**

Only significant comparisons are in bold. Continuous data are reported as median (IQR)

*MLWH* men living with HIV, *ALMI* appendicular lean mass index, *ALM/W* appendicular lean mass to body weight ratio, *IQR* interquartile range, BMD bone mineral density, *BMI* body mass index, *ART* antiretroviral therapy, *LH* luteinizing hormone, *FSH* follicle-stimulating hormone, *PRL* prolactin, *TT* total testosterone, *E1* estrone, *E2* estradiol, *SHBG* sex hormone-binding globulin, *cFT* calculated free testosterone, *E2/T* estradiol to testosterone ratio

MLWH presenting with sarcopenic obesity at ALMI were younger than those without sarcopenic obesity (*p* = 0.025) and they had a longer duration of infection (*p* = 0.045) (Table 2). Concerning sex steroids, serum E2 (*p* = 0.001), E1 (*p* = 0.005), total T (*p* = 0.017), and cFT (*p* = 0.004) were significantly lower in the subgroup of sarcopenic obesity compared to that without sarcopenic obesity according to ALMI (Table 2). However, the prevalence of hypogonadism did not significantly differ between sarcopenic and non-sarcopenic subgroups (5/35, 14.3% vs 27/272 9.9%; *p* = 0.427). Table 3 details differences in bone and body composition parameters between MLWH with biochemical hypogonadism (i.e. total T < 320 ng/mL and/or cFT < 64 pg/mL) and those eugonadal.

**Table 3:** Differences in bone and body composition parameters comparing MLWH with hypogonadism (i.e. TT < 320 ng/mL and/or cFT < 64 pf/mL) to those eugonadal

	n.v	MLWH with eugonadism	MLWH with hypogonadism	*p*-value
*n 307*		275 (89.6%)	32 (10.4%)	
Age (years)	–	46.7 (42.1–49.6)	49.6 (46.4–50.0)	**0.001**
Bone and body composition parameters				
Lumbar BMD (g/cm^2^)	–	0.96 (0.89–1.03)	1.00 (0.90–1.07)	0.261
Total hip BMD (g/cm^2^)	–	0.77 (0.71–0.86)	0.80 (0.73–0.86)	0.385
Neck hip BMD (g/cm^2^)	–	0.84 (0.77–0.95)	0.91 (0.80–0.98)	0.141
Low BMD, *n* (%)		37 (13.5%)	4 (12.1%)	0.831
Lean mass—arms (kg)	–	6.87 (5.99–7.74)	6.10 (5.68–7.35)	0.061
Lean mass—trunk (kg)	–	2.55 (2.35–2.72)	2.55 (2.33–2.87)	0.680
Fat mass—trunk (kg)	–	8.4 (6.5–11.6)	9.2 (6.8–12.8)	0.239
Lean mass—legs (kg)	–	16.8 (15.2–18.2)	16.4 (14.4–18.0)	0.563
Lean mass—total body (kg)	–	59.1 (46.7–56.7)	52.4 (47.7–57.4)	0.700
Lean mass—total body (%)	–	73.3 (69.6–76.4)	73.3 (70.5–77.3)	0.961
Fat mass—total body (kg)	–	17.2 (13.7–21.0)	18.3 (12.8–21.6)	0.574
Fat mass—total body (%)	< 26 or < 28	23.7 (20.5–27.8)	24.7 (19.7–30.4)	0.965
Fat mass— > 26% in aged 20–39 and > 28% in aged 40–50, *n* (%)	–	75 (27.3%)	10 (30.3%)	0.713
ALMI (kg/m^2^)	≥7.26	7.68 (7.05–8.28)	7.41 (6.92–8.34)	0.363
Sarcopenia according to ALMI, *n* (%)	–	92 (33.5%)	15 (46.9%)	0.132
Sarcopenic obesity with ALMI, *n* (%)		31 (11.3%)	5 (15.6%)	0.469
ALM/W (%)	≤28.27	32.86 (30.28–34.62)	31.59 (27.60–34.66)	0.085
Sarcopenia according to ALM/W, *n* (%)	–	34 (12.4%)	10 (31.3%)	**0.004**
Sarcopenic obesity with ALM/W, *n* (%)		30 (10.9%)	9 (28.1%)	**0.006**

Only significant comparisons are in bold. Continuous data are reported as median (IQR)

*MLWH* men living with HIV, *ALMI* appendicular lean mass index, *ALM/W* appendicular lean mass to body weight ratio, *IQR* interquartile range, *BMD* bone mineral density, *TT* total testosterone, *cFT* calculated free testosterone

According to ALM/W, BMI and W/H (both *p* < 0.001) were higher in MLWH in patients with sarcopenic obesity, whereas no difference was found for age nor duration of infection (Table 2). The group of sarcopenic obesity considering the ALM/W had lower levels of total T (*p* = 0.001) and cFT (*p* < 0.001), but serum E2 did not significantly differ in this case (Table 2). Accordingly, the prevalence of hypogonadism (i.e. total T < 320 ng/dL and/or cFT < 64 pg/mL) was higher in the subgroup of sarcopenic obesity compared to the subgroup without sarcopenic obesity (9/38, 23.7% vs 23/269, 8.6%; p = 0.004).

We performed a multivariate regression analysis to identify risk factors for sarcopenic obesity. As for sarcopenia, serum E2 below 18 pg/mL was confirmed as a risk factor for sarcopenic obesity both considering ALMI (OR = 5.58; 95%CI 1.40–22.35; *p* = 0.015) and ALM/W (OR = 26.01; 95%CI 3.00–225.08; *p* = 0.003) together with the duration of HIV (ALMI: OR = 1.12; 95%CI 1.01–1.24; *p* = 0.039—ALM/W: OR = 1.23; 95%CI 1.07–1.42; *p* = 0.004). cFT entered the model as risk factor only for sarcopenic obesity obtained from ALMI (OR = q.29; 95%CI 1.01–1.63; *p* = 0.038) and BMI entered the model as protective factor only for sarcopenic obesity obtained from ALM/W (OR = 0.62; 95%CI 0.44–0.87; *p* = 0.006); no significance was found for total T, cFT, W/H, and frailty.

**Fig. 1:**
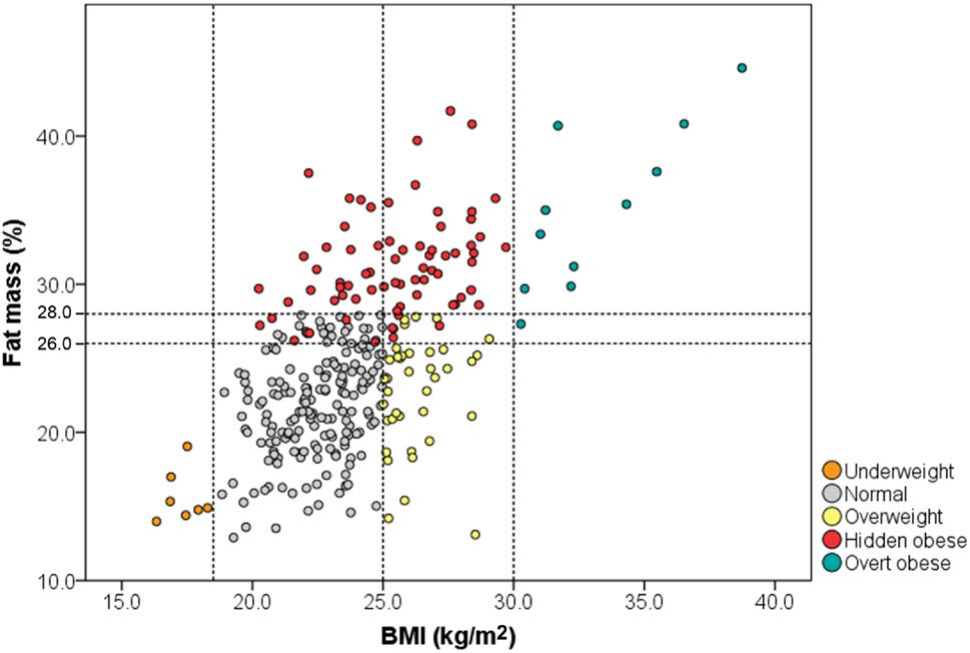
Distribution of the entire cohort according to the BMI and total fat mass percentage.* BMI* body mass index, Each dot corresponds to one subject. Horizontal and vertical dotted lines represent the cut-offs applied for BMI and fat mass percentage to distinguish the abovementioned conditions. Orange: underweight; Grey: normal; Yellow: overweight; Red: hidden obese; Blue: overt obese

The distribution of patients based on body phenotype (defined by BMI and body fat mass) is shown in Fig. 1 in detail 11 patients out of 307 (3.6%) were classified as overt obese, 73 (23.8%) hidden obese, 40 (13.0%) overweight, 175 (57.0%) normal, and 8 (2.6%) underweight.

### Prevalence of low BMD and associated factors

BMD was normal in 264 MLWH (86.0%) and reduced in 43 (14.0%) MLWH (Table 3).

No difference was found for anthropometric variables (BMI, W/H) comparing MLWH with low and normal BMD (Table 4).

**Table 4:** Differences in clinical, biochemical and body composition parameters comparing MLWH low BMD (Z-score ≤ −2.0) to those with normal BMD

	n.v	MLWH with low BMD	MLWH with normal BMD	*p*-value
*n 307*		43 (14.0%)	264 (86.0%)	
Age (years)	–	48.6 (42.9–49.6)	46.8 (42.5–49.7)	0.397
Anthropometric variables				
BMI (kg/m^2^)	18.5–25	23.2 (20.8–25.0)	23.7 (22.1–25.5)	0.058
W/H circumference ratio	< 0.95	0.95 (0.88–0.98)	0.95 (0.90–0.99)	0.654
HIV parameters				
HIV duration (years)	–	16.4 (8.4–24.2)	16.0 (8.5–23.7)	0.993
ART exposure (years)	–	12.9 (6.0–18.9)	14.7 (67.4–20.2)	0.576
Nadir CD4 (cells/μL)	–	274 (90–300)	250 (113–349)	0.764
Current CD4 (cells/μL)	> 400	580 (533–753)	623 (504–831)	0.891
Hormonal measurements				
LH (mIU/mL)	1.4–8.9	4.3 (3.5–6.1)	4.9 (3.5–6.9)	0.420
FSH (mIU/mL)	1.7–6.9	5.2 (3.2–7.2)	5.7 (3.9–8.5)	0.075
PRL (ng/mL)	2.1–17.7	7.0 (5.0–8.7)	7.4 (5.5–10.3)	0.092
Serum E2 (pg/mL)	< 50	22.5 (17.7–27.6)	24.9 (20.2–31.6)	0.054
Serum E1 (pg/mL)		27.3 (15.1–34.5)	28.2 (19.1–37.7)	0.234
Serum TT (ng/dL)	> 320	648 (543–772)	633 (499–800)	0.947
E2/TT	–	0.031 (0.026–0.040)	0.038 (0.031–0.047)	**0.023**
SHBG (nmol/L)	13.5–71.4	50.4 (36.1–65.4)	47.6 (34.6–65.0)	0.717
cFT (pg/mL)	> 64	102 (88–127)	107 (85–133)	0.566
DHT (pg/mL)	165–679	336 (271–421)	382 (263–543)	0.357
Biochemical measurements				
Fasting glucose (mg/dL)	70–100	92 (87–97)	93 (87–98)	0.495
Insulin (mIU/mL)	2–23	7.2 (4.6–12.0)	7.6 (5.1–12.6)	0.490
HOMA index	< 2.5	1.75 (0.99–3.00)	1.70 (1.15–3.00)	0.478
HbA1c (mmol/mol)	20–38	34.0 (31.0–37.0)	34.0 (31.0–36.6)	0.797
Total cholesterol (mg/dL)	< 200	200 (172–224)	187 (162–213)	0.092
LDL cholesterol (mg/dL)	< 100	126 (100–144)	117 (96–141)	0.203
HDL cholesterol (mg/dL)	> 45	49 (37–58)	45 (37–53)	0.251
Triglycerides (mg/dL)	< 180	136 (84–226)	129 (88–188)	0.705
Serum calcium (mg/dL)	8.5–10.5	9.5 (9.2–9.7)	9.4 (9.2–9.6)	0.086
Corrected calcium (mg/dL)	8.5–10.5	9.0 (8.8–9.2)	8.9 (8.7–9.1)	0.386
Serum phosphorus (mg/dL)	2.5–5.1	3.2 (2.8–3.7)	3.0 (2.7–3.4)	0.098
Serum Ca/P	–	3.03 (2.70–3.32)	3.08 (2.76–3.49)	0.407
Serum magnesium (mg/dL)		2.12 (2.03 (2.15)	2.07 (1.97–2.15)	0.356
Albumin (g/dL)	3.5–5.0	4.6 (4.5–4.8)	4.6 (4.4–4.7)	0.190
PTH (pg/mL)	15–88	30.2 (22.0–40.5)	33.1 (29.6–48.0)	0.177
25OH-vitamin D (ng/mL)	30–100	27.6 (21.5–39.3)	27.9 (21.1–34.5)	0.432
Total alkaline phosphatase (U/L)	38–126	72 (56–86)	73 (60–93)	0.238
Osteocalcin (ng/mL)	6.5–42.3	18.3 (14.7–21.8)	18.6 (16.1–21.4)	0.622
CTX (ng/mL)	0.034–0.635	0.53 (0.34–0.72)	0.52 (0.37–0.75)	0.757
Lifestyle and drug use				
Smoking	–	16 (37.2%)	105 (40.2%)	0.708
Alcohol use (moderate/intense)	–	21 (48.8%)	143 (54.6%)	0.484
Opioids use	–	8 (18.6%)	56 (21.4%)	0.679
Comorbidities and frailty				
Diabetes	–	0 (0%)	7 (2.7%)	0.278
Hypertension	–	4 (9.3%)	49 (18.7%)	0.132
Cardiovascular disease	–	2 (4.7%)	17 (6.5%)	0.644
Dyslipidemia	–	20 (46.5%)	129 (49.4%)	0.723
HCV/HBV co-infection	–	7 (16.3%)	50 (19.0%)	0.670
Previous atraumatic fractures	–	2 (4.7%)	3 (1.1%)	0.093
Frailty index	< 0.21	0.25 (0.20–0.33)	0.24 (0.17–0.32)	0.992
Lumbar BMD (g/cm^2^)	–	0.82 (0.77–0.84)	0.98 (0.92–1.05)	** < 0.001**
Total hip BMD (g/cm^2^)	–	0.75 (0.63–0.83)	0.86 (0.79–0.97)	** < 0.001**
Neck hip BMD (g/cm^2^)	–	0.69 (0.61–0.75)	0.79 (0.73–0.87)	** < 0.001**
Lean mass—arms (kg)	–	6.49 (5.53–7.38)	6.88 (5.97–7.73)	0.036
Lean mass—trunk (kg)	–	2.35 (2.15–2.55)	2.56 (2.38–2.73)	** < 0.001**
Fat mass—trunk (kg)	–	7.5 (6.3–11.3)	8.5 (6.6–11.6)	0.177
Lean mass—legs (kg)	–	15.8 (14.4–17.5)	16.9 (15.4–18.3)	**0.009**
Lean mass—total body (kg)	–	50.1 (45.6–54.4)	53.4 (50.1–57.3)	**0.001**
Lean mass—total body (%)	–	72.4 (66.0–75.2)	73.6 (70.0–77.3)	0.135
Fat mass—total body (kg)	–	15.1 (13.1–20.6)	17.4 (13.8–21.6)	0.177
Fat mass—total body (%)	< 26 or < 28	24.0 (21.4–27.8)	23.8 (20.0–27.9)	0.723
Fat mass— > 26% in aged 20–39 and > 28% in aged 40–50, n (%)	–	37 (86.0%)	202 (76.5%)	0.163
ALMI (kg/m^2^)	≥7.26	7.54 (7.03–8.15)	7.68 (7.06–8.29)	0.363
Sarcopenia according to ALMI, *n* (%)	–	16 (37.2%)	91 (34.2%)	0.703
ALM/W (%)	≤28.27	33.46 (30.73–35.77)	32.53 (30.09–34.50)	0.163
Sarcopenia according to ALM/W, *n* (%)	–	6 (14.6%)	37 (14.0%)	0.916

Only significant comparisons are in bold. Continuous data are reported as median (IQR)

*MLWH* men living with HIV, *IQR* interquartile range, *BMI* body mass index, *ART* antiretroviral therapy, *LH* luteinizing hormone, *FSH* follicle-stimulating hormone, *PRL* prolactin, *TT* total testosterone, *E1* estrone, *E2* estradiol, *SHBG* sex hormone-binding globulin, *cFT* calculated free testosterone, *E2/T* estradiol to testosterone ratio, *SHBG* sex hormone-binding globulin, *Ca/P* calcium to phosphorus ratio, *DXA* dual-energy X-ray absorptiometry

Among body composition parameters lean mass significantly differed in relation to BMD values. Indeed, lean mass assessed at trunk (*p* < 0.001), arms (*p* = 0.036), legs (*p* = 0.009), and total body (*p* = 0.001) was significantly lower in the subgroup of low BMD (Table 4); no difference was found for fat mass at any site. Furthermore, BMD values (lumbar, total hip and neck hip) were significantly associated with lean and fat mass assessed at total and trunk levels (*p* range: < 0.001–0.025) (Table 5).

**Table 5:** Bivariate linear regression analysis among BMD values at lumbar and hip (total or neck) sites and body composition parameters regarding lean and fat mass

	Lumbar BMD			Total hip BMD			Neck hip BMD		
	*p-value*	*R*^*2*^	*β*	*p-value*	*R*^*2*^	*β*	*p-value*	*R*^*2*^	*β*
Total lean mass	** < 0.001**	0.093	0.306	** < 0.001**	0.176	0.419	** < 0.001**	0.139	0.373
Total fat mass	**0.001**	0.033	0.181	** < 0.001**	0.057	0.254	**0.014**	0.020	0.140
Trunk lean mass	** < 0.001**	0.077	0.277	** < 0.001**	0.106	0.324	** < 0.001**	0.079	0.281
Trunk fat mass	**0.002**	0.030	0.174	** < 0.001**	0.051	0.226	**0.025**	0.017	0.129

Among serum sex steroids only the E2/T ratio was significantly lower in MLWH with low BMD compared to those with normal BMD (*p* = 0.023) (Table 4). However, at bivariate linear regression, no significant association was found for E2/T and BMD at any site.

Concerning other biochemical measurements related to mineral homeostasis, lifestyle habits, and HIV variables, parameters did not differ between low BMD and normal BMD subgroups, as well as lifestyle habits (Table 4).

At multivariate logistic regression analysis performed to identify risk factors for low BMD, the co-variates were chosen including variables that at univariate analysis displayed *p*-value < 0.10. This multivariate analysis showed that serum E2 below 18 pg/mL was the only independent risk factor for low BMD (OR = 3.06; 95%CI 1.03–9.07; *p* = 0.043). On the contrary, SHBG, total T and E2/T ratio, years of HIV, and sarcopenia lost the statistical significance.

## Discussion

The current study provides evidence that, notwithstanding the notable improvement in medical conditions of this population allowed by novel therapies, loss of muscle mass and low BMD remain frequent findings in young adult MLWH. Furthermore, among sex steroids, both T and E2 are confirmed as important determinants of body composition in MLWH also since they resulted independently associated with sarcopenia and low BMD, respectively. These results corroborate our previous data concerning the strict connection between estrogens, body fat, and health status in MLWH [25]. In parallel, this is among the first studies investigating the distribution of obesity phenotypes and the prevalence of sarcopenic obesity among MLWH showing that sarcopenic obesity is present in 11–12% of enrolled MLWH regardless of the definition used, but deep differences emerged using two different diagnostic definitions. Considering the amount of fat mass, up to one forth of our cohort presents with hidden obesity, demonstrating a high prevalence of this condition in MLWH < 50.

Over the last decade, sarcopenia in MLWH drew the attention of researchers in this field. However, the low agreement between methods to assess and define this disease turn it challenging to compare different studies, both in MLWH and in the general population. Several operational definitions are used to diagnose sarcopenia and pre-sarcopenia, some including functionality analysis of muscle strength (EWGSOP2) [46] but not others (Baumgartner’s criteria) [23, 35]. Beyond this limitation in the diagnostic criteria, other factors make the prevalence of sarcopenia in MLWH heterogenous across studies: for instance, lean mass can be assessed by DXA or electrical bioimpedance (BIA), and different methods are used to assess physical performance. In our study the prevalence of sarcopenia significantly changed according to two different criteria used, being higher with ALMI than ALM/W. The 34.9% prevalence of sarcopenia we obtained by ALMI is slightly higher compared to previous studies [47–52] that used lean body mass as the only parameter to define sarcopenia, as in our case, with a pooled prevalence of 28.8% according to a recent metanalysis [37]. Otherwise, the 14.3% prevalence we obtained by ALM/W was lower than previous studies, but in line with a recent one showing a prevalence of 11.8% [53]. Differences between ALMI and ALM/W definitions of sarcopenia might be due to a different accuracy in identifying sarcopenia according to i) HIV status (MLWH *vs* uninfected men, ii) different phenotypes of sarcopenia (e.g. low muscle mass *plus* high BMI vs low muscle mass plus low BMD). Accordingly, in our study ALMI seems to better identify sarcopenic MLWH with low muscle and bone mass (osteosarcopenia), while ALMI/W better identify patients with low percentage of lean mass and increased percentage of fat mass (i.e. the sarcopenic obesity). Conversely, ALMI is most often used in HIV setting [37], while ALM/W is mainly used in HIV uninfected men [23, 24]. Criteria used for defining sarcopenia by anthropometry should be preferred in young to middle-aged men as in this age range muscle strength is rarely impaired. Accordingly, there is uncertainty about the use of muscle strength in sarcopenia diagnosis in MLWH and its use in young men may lead to underestimation of sarcopenia. Overall, studies that considered only muscle mass as criterion to define sarcopenia found higher prevalence compared to those that added muscle strength or muscle function [19, 37, 54]. Since sarcopenia is a recognized predictor of all-cause mortality [55, 56], frailty and increased hospitalization costs [57], this is a relevant finding that may have major implications for those aging with HIV and should be considered in raising awareness about this aspect among HIV health professionals.

In this study both serum total T and cFT were significantly lower in sarcopenic MLWH independently from the criterion used to define sarcopenia suggesting a potential role of T in ensuring adequate lean mass (both muscle and bone mass). This is in line with well-known physiological anabolic actions of androgens on body composition by acting directly on muscle and through their conversion to estrogens on bone [15, 16, 58, 59].

Although it has been estimated that MLWH presented sixfold greater odds of sarcopenia compared with matched controls of same age [37], sarcopenia remains largely underdiagnosed in the clinical care of MLWH. Indeed, HIV has been not yet identified as a contributing factor in the sarcopenia consensus and guidelines [46].

In this study the distribution of obesity phenotypes (overt, hidden, and apparent obesity) are quite different from those reported in a recent study among Japanese MLWH, with hidden obesity being detected in 46% of Konishi’s cohort and in 24% of our cohort [60]. Differences can be explained by the older age of the Asiatic cohort compared to ours and by the different cut off for fat mass used to define obesity (≥ 20% in Konishi et al. vs ≥ 26–28% in our study), as should be done in relation to patients’ race [23]. On the other hand, in the literature little is currently known about the prevalence of sarcopenic obesity among MLWH. In our MLWH cohort we found an overall prevalence of sarcopenic obesity of 11–12% regardless of the diagnostic criterion used for sarcopenia. Independently from the criteria used for defining sarcopenia, serum TT and cFT were both lower in MLWH with than in those without sarcopenic obesity confirming the importance of sex steroids in sarcopenic obesity [61]. Again, as discussed above for sarcopenia, sarcopenic obesity is also associated with a vast array of adverse health outcomes, including morbidity and mortality in older population without HIV [61, 62]; MLWH may experience many of these consequences more often than people aging without HIV. As such, sarcopenic obesity is expected to pose a major socioeconomic burden for MLWH. Thus, sarcopenic obesity diagnosis and development of intervention programs to prevent/treat this condition in MLWH are advocated.

**Fig. 2:**
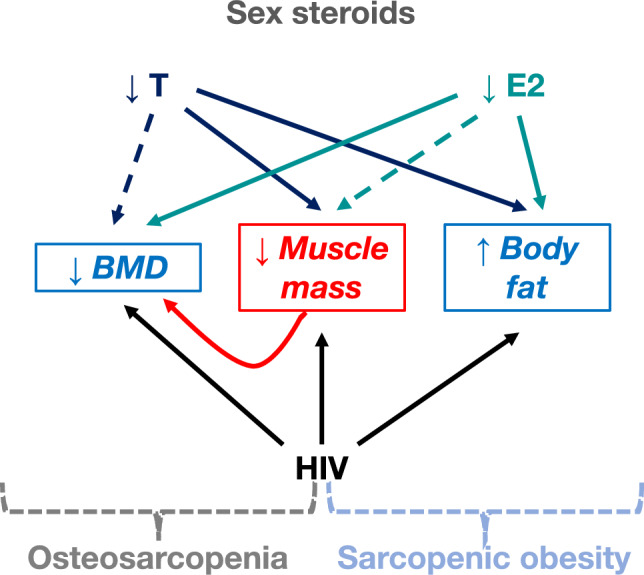
Hypothesis of interactions connecting sarcopenia, low BMD, body fat and sex steroids in MLWH *T* testosterone, *E2* estradiol. Continuous lines represent the strongest associations, dotted lines represent the weakest ones

Strictly associated with the presence of sarcopenia is the finding of impaired BMD. Indeed, we demonstrate that low BMD defined as *Z*-score below −2 is highly prevalent among MLWH younger than 50, confirming data available in literature [63, 64]. Similar considerations that we made for loss of muscle mass can be drawn for low BMD, since these two conditions are strictly related. Accordingly, lean mass was significantly lower in MLWH with low than in those with normal BMD and lean mass was directly related to BMD in this study, confirming data available in literature in MLWH [51, 53] and general population [65]. Bone and muscle, in fact, closely interact with each other not only anatomically, but also chemically and metabolically. From a clinical standpoint, osteoporosis as well as sarcopenia is linked to negative outcomes such as falls, fractures, loss of function, frailty, and mortality increase, thus generating significant personal and socio-economic costs. Therefore, it has been proposed that when BMD loss is synchronic with decreased muscle mass, it should be interpreted as a single diagnosis of osteosarcopenia, which may be preventable and treatable [66]. This study first highlights and confirms the central role of sex steroids, estrogens rather than androgens, in the strict interconnection between bone, muscle, and adipose tissue. MLWH with low serum E2 (< 18 pg/mL) had threefold increased likelihood to develop sarcopenia and/or low BMD. The role of estrogen deficiency on low BMD has been previously demonstrated by our group in MLWH [63] as well as in male population [17, 67]. Furthermore, even a disproportional estrogens-androgens balance was found to be involved in the muscle and BMD loss, since MLWH with sarcopenia and with low BMD had lower E2/T ratio compared to non-sarcopenic and normal BMD subgroups, respectively. These findings reiterate our previous data adding further value to the deep involvement of sex steroids, particularly estrogens, in the vicious circle connecting bone, fat, and skeletal muscle [25] (Fig. 2). As a confirmation of the protective role of estrogens against sarcopenia and BMD loss, sex differences in muscle and strength loss in HIV cohorts have been already reported [52, 68–70]. At present, data about the role of E2 in the homeostasis of musculoskeletal system in males are not only few, but also conflicting; hence, estrogen deficiency in men and its consequences on muscle represents still a challenge both in research and clinical settings. Overall, literature speaks in favor of a direct relationship between serum E2 and muscle mass in men [71, 72] with a minority of studies showing opposite results [73].

Different speculations can be made to clarify pathophysiological mechanisms leading to the persistence of sarcopenia/osteoporosis in HIV setting nowadays. Fat infiltration, typical of both primary sarcopenia and HIV-related fat alterations, is associated to increased metabolic and immunological activity of the adipose tissue [18]. On the other hand, it is not clear whether this decrease in muscle and bone mass found in MLWH is different from that occurring in the general population due to aging. Indeed, aging is the primary cause of sarcopenia [46] and osteoporosis among healthy people. Since MLWH enrolled in this study were younger than 50 years, the high prevalence of both sarcopenia and sarcopenic obesity in our cohort supports an accentuated aging in these patients [1, 74, 75]; in addition, the effect of aging is further emerging in MLWH due to the progressive improvement of life expectancy.

Considering metabolic parameters, BMI, insulin levels and HOMA index present different behaviors according to the two classifications of sarcopenia. Indeed, BMI is known to not include information about loss of lean mass. Likewise, in the sarcopenia evaluation, the insulin levels appear to provide no additional information on the metabolic status of the subjects, remaining within the normal ranges in the entire sample. Furthermore, dividing the population according to the definition of sarcopenic obesity, the BMI resulted significantly higher in the group with sarcopenic obesity defined through the ALM/W criteria, while no differences has been described according to the ALMI formula. Lastly, sarcopenic obesity does not determine changes in the prevalence of diabetic or cardiovascular disease in our sample, considering both definition methods.

This study has strengths and limitations. This is a properly-designed study to investigate the gonadal function in MLWH in which measurement of total T, E2, DHT, and E1were obtained by the recommended analytical technology (LC–MS/MS) alongside SHBG in order to calculate free T that is recognized to be the most reliable biomarker to detect the gonadal status in these patients [58, 76, 77]. Other points of strength are the detailed study of body composition and the selection of young/middle-aged subjects in our cohort, that allowed us to downgrade and avoid the influence of physiological aging on both T levels and body composition changes. The lack of muscle functionality tests for the diagnosis of sarcopenia and DXA inability to quantify fat infiltration into muscle tissue represent limitations. This study does not allow establishing a cause-effect relationship among all these factors due to its cross-sectional design, but it helps in understanding factors associated to sarcopenia and low BMD; for this reason, the lack of a control group of HIV-uninfected men could be considered as a minor limit.

In conclusion, this study shows that sarcopenia and low BMD are common findings among MLWH younger than 50 years. Additionally, the prevalence of sarcopenic obesity and hidden obesity is high and it may be overlooked by weight measurement alone or BMI calculation with a consequent undermanagement of the increased cardiometabolic risk. Within the multidimensional network of factors leading to reduced BMD and lean mass, an imbalanced E2/TT ratio and low serum E2 (< 18 pg/mL) seem to be more relevant rather than TT alone.

In clinical practice the use of different criteria for the definition of sarcopenia (i.e. ALMI and ALM/W) in the setting of MLWH probably allows identifying different forms of sarcopenia since ALMI better characterize osteosarcopenia, while ALM/W leads to identification of sarcopenic obesity. Finally, the recognition of MLWH as vulnerable population who would benefit from an early diagnosis of sarcopenia is advocated for the clinical practice and future guidelines.

## Data Availability

The datasets generated during and/or analysed during the current study are available from the corresponding author on reasonable request.
